# Experience and clinical efficacy of gut‐directed hypnotherapy in an Asian population with refractory irritable bowel syndrome

**DOI:** 10.1002/jgh3.12770

**Published:** 2022-05-19

**Authors:** Ayodele Sasegbon, Syed S Hasan, Peter J Whorwell, Dipesh H Vasant

**Affiliations:** ^1^ Neurogastroenterology Unit, Wythenshawe Hospital Manchester University NHS Foundation Trust Manchester UK; ^2^ Division of Diabetes, Endocrinology and Gastroenterology University of Manchester Manchester UK

## Abstract

**Background and Aim:**

Recent studies have highlighted the high worldwide prevalence of irritable bowel syndrome (IBS) and disparities in its management between ethnic groups. For instance, gut‐directed hypnotherapy (GDH), one of the most effective evidence‐based treatments for IBS, is not recommended in Asian countries partly due to lack of population‐specific outcome data. In this context, we evaluated the outcomes of GDH in an Asian population.

**Methods:**

Consecutive British Asian patients with refractory IBS who received 12‐sessions of GDH using the Manchester protocol were included. Patients were treated by a team including a therapist able to speak several Asian languages. All patients prospectively completed the following questionnaires before and after GDH: IBS symptom severity score (IBS‐SSS), hospital anxiety and depression scale (HADS), non‐colonic symptom score, and the quality‐of‐life (QOL) score. The primary outcome measure was response to GDH defined by ≥50‐point reduction in IBS‐SSS. Pre‐ and post‐treatment data were compared statistically.

**Results:**

Forty‐four Asian patients with IBS (age 49 ± 13 years; 29 [66%] female; baseline IBS‐SSS: 332.8 ± 94.6) completed GDH. Overall, 37 of 44 (84%) achieved a ≥50‐point reduction in IBS‐SSS and 25 of 44 (57%) achieved ≥30% reduction in abdominal pain scores. Following GDH, there were also significant mean improvements in IBS‐SSS (−132.1, *P* < 0.0001), non‐colonic symptom score (*P* < 0.0001), QOL score (*P* < 0.0001), HADS‐anxiety (*P* < 0.0001), and HADS‐depression (*P* < 0.0001), compared with baseline.

**Conclusion:**

Regardless of the ethnicity of the therapist, GDH was highly effective with similar response rates to outcomes in other IBS populations, supporting the development of GDH in Asian countries.

## Graphical abstract

This study suggests that hypnotherapy is a highly effective treatment approach in an Asian population with refractory irritable bowel syndrome. Hypnotherapy significantly improves gastrointestinal, extra‐intestinal, psychological symptoms, and quality of life. These data have highlighted the need for developing hypnotherapy services and population‐specific clinical studies to confirm these findings in Asian countries.

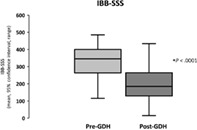

## Introduction

Irritable bowel syndrome (IBS) is classed as a disorder of gut–brain interaction,^1^ based upon advances in the understanding of its pathophysiological basis. Aberrant communication between the enteric and central nervous systems leads to impaired gut sensory and motor function resulting in the cardinal symptoms of altered bowel habit, abnormal stool consistency, and abdominal pain.^2^, ^3^, ^4^ There is increasing recognition of the multifactorial nature of IBS and the importance of complex interactions between patients: the communities and societies of which they are part, the gut microbiome,^5^ their propensity towards anxiety,^6^ their genetic makeup,^7^, ^8^ and gastrointestinal insults such as gastroenteritis.^9^

Recent global epidemiological studies have shown that the prevalence of IBS varies from country to country in a manner thought to be due to cultural stigma and perception from both patients and health professionals.^10^, ^11^, ^12^ Furthermore, within countries, studies have shown interethnic differences in IBS prevalence.^13^ With respect to the diagnosis and management of IBS, differences have also been shown to exist between ethnic groups.^14^ While these observed differences in the management of IBS may be unavoidable due to cultural differences in the expectations patients from different ethnic groups have of their health professionals and vice versa, there remains a concern that poor communication or inequity within health organizations and wider society may be contributing factors.^15^

Conventional, primarily biological approaches to IBS, with medications and dietary modification, can be effective,^16^, ^17^, ^18^ but despite this, up to 25% of patients do not improve despite maximal medication‐based therapy.^19^ Moreover, the acceptability and practicalities of dietary modification for IBS may also vary between countries, and may be difficult in some South Asian countries due to regional food preferences, cultural factors, and the frequency of lactose malabsorption.^20^ Furthermore, it has long been thought that a purely biological treatment approach may be insufficient to holistically manage patients with IBS, particularly in the most treatment‐resistant cases. Indeed, there is increasing evidence to support the view that optimal management involves integrated, multidisciplinary care within a biopsychosocial framework including gut–brain behavioral therapies.^21^, ^22^, ^23^, ^24^

Gut‐directed hypnotherapy (GDH),^25^ a brain‐gut behavioral therapy, is one of the few treatments that has been proven to have efficacy in patients with refractory IBS.^26^ Multiple studies over three decades have shown that GDH is effective at treating IBS in both adults^25^, ^26^, ^27^ and children.^28^, ^29^ It is thought to exert its effect by reducing visceral hypersensitivity, modulating the cortical processing of painful gastrointestinal stimuli^25^, ^30^ and ameliorating psychologically mediated alterations to gastrointestinal motility.^25^ Despite GDH's large and increasing evidence base, studies of GDH performed thus far have been performed in predominantly Caucasian populations. While useful, it is not currently known how effective GDH is at treating IBS within other ethnic groups, each with different conceptions of illness and attitudes toward health professionals. In the absence of population‐specific data, GDH is therefore not currently recommended in Asian countries.^31^ However, the unmet need to develop non‐pharmacological treatments for IBS in Asian countries has recently been highlighted.^32^

In order to understand whether or not GDH might be effective in Asian populations with IBS, the United Kingdom, a country with a diverse multi‐ethnic population, including a large Asian population,^33^ is a good starting point. In this context, for the first time, we evaluated outcomes from consecutive patients of Asian origin that had undergone GDH in a tertiary referral center in Manchester, United Kingdom, to understand the potential of this treatment to be developed in Asian countries.

## Methods

### Aims

The study aimed to:

- evaluate the effectiveness of GDH in treating IBS in a British Asian population;
- compare GDH treatment response rates between Asians of Indian and Pakistani heritage;
- compare response rates to GDH between patients treated by Asian and non‐Asian therapists.

### Design and patient population

In a service evaluation of the effectiveness of GDH in clinical practice, consecutive patients of Asian descent who underwent GDH at the Hypnotherapy Unit at Wythenshawe Hospital, Manchester University NHS Foundation Trust, United Kingdom, between 2017 and 2021, were identified from a prospectively maintained database. As this was an evaluation of outcomes from the existing service, ethical approval was not required. All patients included were ≥18 years of age and met the National Institute for Health and Care Excellence (NICE) and British Society of Gastroenterology (BSG) criteria for a diagnosis of severe, refractory IBS, to qualify for GDH. This meant patients had to have exhausted medical and dietary therapies over a 12‐month period.^4^ IBS was sub‐typed according to Rome IV criteria using the Bristol Stool Chart (constipation predominant [IBS‐C], diarrhea predominant [IBS‐D], and mixed [IBS‐M]).^34^ Exclusion criteria included: the presence of other gastrointestinal pathology and the presence of non‐gastrointestinal diseases sufficiently severe as to render patients clinically unstable or to have a large deleterious impact on their quality of life.

### Outcome measures

As in our previous studies, outcome measures were questionnaire‐based, involving observed changes between pre‐ and post‐GDH assessments using a series of validated instruments.^27^, ^28^

The primary outcome measures were clinical response, defined as a 50‐point improvement in the IBS symptom severity score (IBS‐SSS) after GDH. Secondary outcome measures included the Food and Drug Administration (FDA) recommended outcome measure for IBS interventions of >30% improvement in abdominal pain scores.^35^ Other secondary outcome measures included the hospital anxiety and depression scale (HADS), quality‐of‐life (QOL) score, and non‐colonic symptom score (NCSS).

### IBS symptom severity score

The IBS‐SSS is a validated questionnaire that assesses the multifactorial negative impact of IBS on the lives of individual patients based on their responses to questions that fall into five broad categories.^36^ These include intensity of pain, frequency of abdominal pain, severity of abdominal distension, satisfaction with current bowel habit, and how their symptoms affect their QOL.

The IBS‐SSS is scored out of 500 (100 points per category). Previous studies have shown that a reduction of ≥50 points constitutes a significant clinical improvement.^36^ As in previous studies, a ≥50‐point improvement was the definition of clinical response to GDH. The secondary outcome measure of ≥30% improvement in abdominal pain response rate post‐GDH was calculated from the combined abdominal pain frequency and intensity sub‐scores of IBS‐SSS as in previous studies.^27^, ^28^, ^37^, ^38^

### Non‐colonic symptom score

The NCSS is a questionnaire developed by Gonsalkorale *et al*.^39^ in 2002. It is designed to assess the relative burden of extra‐intestinal symptoms on the lives of individuals with IBS. Reduction in this symptomatic burden has been shown to lead to improvements in patients' reported quality of life.^27^ Patients are instructed to score the impact 10 different extra‐intestinal symptoms have in their lives, with each symptom being scored out of 100. Symptoms are headaches, backache, thigh pain, other bodily aches, lethargy, nausea and or vomiting, early satiety, heartburn, flatulence, and urinary symptoms. The total score is then divided by two to give a score out of 500.

### Quality of life

The IBS QOL questionnaire was developed by Gonsalkorale *et al*. in 2002.^39^ It is comprised of a total of 15 questions organized into five categories. The categories and questions are psychic well‐being (ability to cope with problems, confidence, and perceived security), physical well‐being (sleep quality, sense of physical well‐being), mood (magnitude of irritability, extent of worrying, feelings of hopefulness, enjoyment of life), locus of control (feelings of helplessness, ease of decision‐making), social/relationship (relationships with close family, maintenance of friendships, sense of inferiority, feeling wanted/needed, enjoyment of leisure).^39^ Each question is scored out of 100, and the total score is divided by 3 to give a value out of 500. The higher the score, the better the patient's perceived QOL.

### Hospital anxiety and depression scale

The HADS is a long‐used questionnaire^40^ consisting of two broad domains: anxiety and depression. Within each domain are seven questions, each scored out of three. Therefore, each domain has a total score of 21. Scores ≥10 in each domain have been shown to indicate clinical levels of depression and anxiety.^27^

### Gut‐directed hypnotherapy

Each patient received a 1‐h GDH session on a one‐to‐one basis, weekly for a total of 12 weeks. GDH was performed by experienced hypnotherapists, one of whom was South Asian and able to speak Hindi and Urdu. GDH was performed using the Manchester protocol, which has been described in detail elsewhere.^27^, ^28^ All patients completed questionnaires before and after the full 12‐week course of GDH.

### Statistical analysis

Questionnaire data were expressed as mean ± SD unless stated otherwise. Pre‐ and post‐GDH data for each questionnaire (IBS‐SSS, NCSS, QOL, HADS) were compared using Student's paired *t*‐tests. Fishers exact test were used to compare GDH response rates between British Asians of Pakistani and Indian heritage and treatment response rates between patients treated by Asian or non‐Asian hypnotherapists, as well as those who were known first‐generation British Asians compared with those who were not first‐generation Asians. Data were analyzed using SPSS (IBM SPSS Statistics 22.0; IBM, Armonk, NY, USA).

## Results

Forty‐four Asian patients with IBS (age 49 ± 1.9 years; 29 [66%] female) received 12 sessions of GDH. Fifteen patients were of Indian heritage, 25 of Pakistani heritage, 2 of Arabic heritage, and 2 of other Middle Eastern heritage. Eleven patients were known first‐generation British Asians, whereas 21 patients were born in the United Kingdom, and the place of birth was unknown for 11 patients. Twenty‐two patients had IBS‐D, 15 IBS‐C, and 7 IBS‐M. The mean duration of IBS was 10 ± 6 years. Forty of forty‐four (91%) received GDH in English, while four of forty‐four (9%) received GDH in Urdu.

### IBS symptom severity

Clinical response defined by a ≥50‐point improvement in IBS‐SSS from baseline was achieved in 37 patients (84%) after GDH. Of these, 26 (59%) patients achieved the more demanding endpoint of a ≥100‐point improvement, 21 (48%) a ≥150‐point improvement, and 15 (34%) a ≥200‐point improvement in IBS‐SSS.

**Figure 1:**
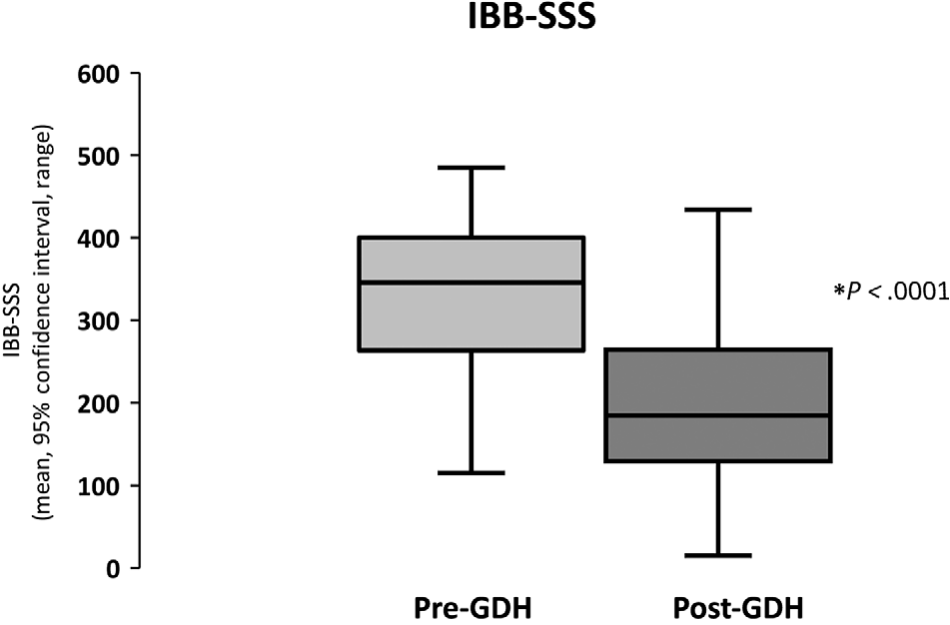
Improvement in irritable bowel syndrome symptom severity score (IBB‐SSS) post‐gut‐directed hypnotherapy (GDH).

Overall, there was a significant improvement in mean IBS‐SSS (baseline 332.75 ± 94.62 *vs* post‐GDH 200.66 ± 104.23; T (43) = 7.87, *P* < 0.0001; Fig. 1, Tables 1 and 2). Twenty‐five of 44 patients (57%) achieved a ≥30% improvement in abdominal pain scores.

**Table 1:** Mean and percentage change in outcome measures to gut‐directed hypnotherapy in British Asians with severe refractory irritable bowel syndrome (IBS)

Outcome measures	Mean change pre‐ and post‐GDH (±SD)	Mean % change pre‐ and post‐GDH (±SD)	*P* values pre‐ and post‐GDH
IBS‐SSS	−132.1 (±111.3)	−36.1 (±39.8)	**<0.0001**
NCSS	−74.5 (±75.0)	−26.0 (±41.3)	**<0.0001**
QOL	+74.8 (±104.2)	+79.0 (±189.0)	**<0.0001**
HADS (anxiety)	−3.8 (±4.1)	−26.8 (±29.6)	**<0.0001**
HADS (depression)	−2.9 (±3.0)	−30.5 (±31.1)	**<0.0001**

GDH, gut‐directed hypnotherapy; HADS, hospital anxiety and depression scale; IBS‐SSS, IBS symptom severity score; NCSS, non‐colonic symptom score; QOL, quality‐of‐life.

The values that are in bold in the tables are statistically significant (i.e. have *P* values < 0.05).

**Table 2:** Mean irritable bowel syndrome (IBS) symptom severity sub‐scores for all outcome measures before and after hypnotherapy in British Asians with severe refractory IBS

Questionnaire	Pre‐GDH	Post‐GDH	*P* value
Total IBS symptom severity score			
Intensity of abdominal pain	55.68 ± 28.80	28.48 ± 25.95	**<0.0001**
Frequency of abdominal pain	59.77 ± 35.21	37.27 ± 30.92	**<0.0001**
Abdominal distension	63.86 ± 24.29	33.95 ± 23.84	**<0.0001**
Satisfaction with bowel habit	74.64 ± 24.15	47.64 ± 24.72	**<0.0001**
Impact of IBS on QOL	78.80 ± 16.80	53.32 ± 24.41	**<0.0001**
Total non‐colonic symptom score			
Nausea/vomiting	30.39 ± 28.90	18.65 ± 23.96	**0.0007**
Early satiety	28.75 ± 27.40	18.95 ± 21.99	**0.0059**
Headaches	55.43 ± 31.15	42.26 ± 28.92	**0.0020**
Backaches	54.36 ± 30.52	42.47 ± 29.25	**0.0056**
Lethargy	76.48 ± 24.73	54.53 ± 27.77	**<0.0001**
Flatulence	65.45 ± 29.62	49.09 ± 27.73	**0.0021**
Heartburn	37.25 ± 28.74	26.47 ± 24.52	**0.0007**
Urinary symptoms	53.77 ± 33.51	42.23 ± 29.92	**0.0302**
Thigh pain	38.66 ± 35.16	25.35 ± 29.23	**0.0002**
Musculoskeletal pain	58.52 ± 33.39	38.72 ± 29.81	**0.0002**
Total QOL			
Ability to cope with problems	40.84 ± 24.04	60.58 ± 21.88	**<0.0001**
Confidence and security	33.66 ± 23.34	60.39 ± 22.35	**<0.0001**
Quality of sleep	40.80 ± 23.30	55.40 ± 23.16	**<0.0001**
Physical well‐being	35.50 ± 22.97	58.00 ± 19.12	**<0.0001**
Irritability	39.93 ± 25.10	48.35 ± 23.70	0.1814
Worrying	36.16 ± 30.45	42.49 ± 22.81	0.2353
Hopefulness	40.84 ± 23.24	61.05 ± 23.28	**0.0001**
Enjoyment of life	37.39 ± 22.62	55.74 ± 19.39	**<0.0001**
Helplessness	35.70 ± 23.26	55.72 ± 21.59	**<0.0001**
Decision‐making	51.89 ± 29.05	59.28 ± 25.79	0.8334
Relationships with family	50.41 ± 23.38	68.95 ± 18.89	**<0.0001**
Maintenance of friendships	55.20 ± 28.73	71.19 ± 21.85	**0.0004**
Inferiority	51.57 ± 26.80	61.60 ± 25.21	**0.0091**
Feeling wanted	51.80 ± 28.55	62.72 ± 26.35	0.0557
Enjoyment of leisure	38.34 ± 23.68	53.77 ± 23.07	**0.0015**

GDH, gut‐directed hypnotherapy; QOL, quality‐of‐life.

The values that are in bold in the tables are statistically significant (i.e. have *P* values < 0.05).

### Extra‐intestinal symptom severity

**Figure 2:**
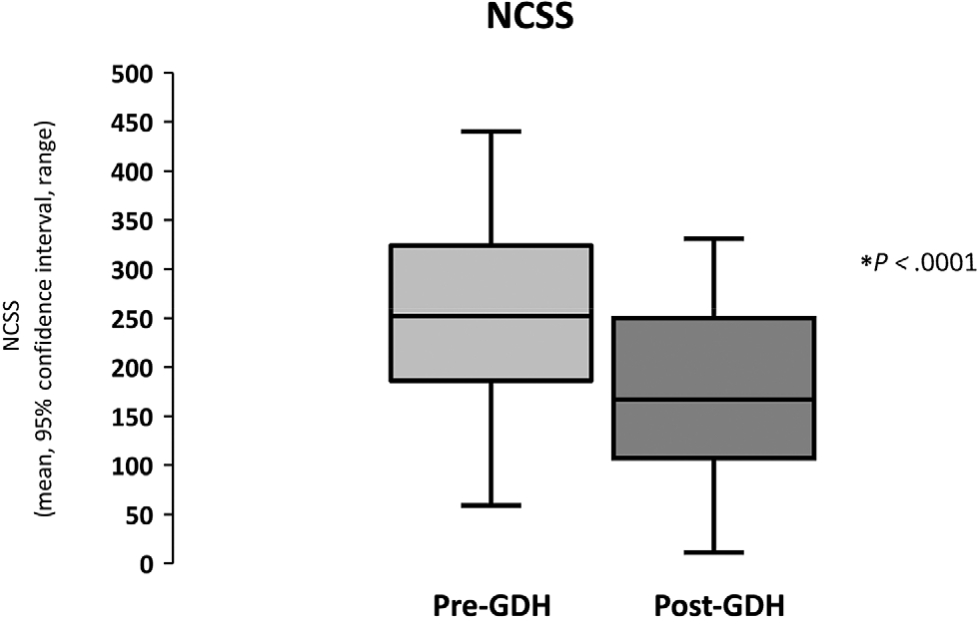
Improvement in non‐colonic symptom score (NCSS) post‐gut‐directed hypnotherapy (GDH).

There were highly significant improvements in the severity of all of the somatic extra‐intestinal symptoms of IBS following GDH in this population (Table 2) and a significant overall improvement in mean NCSS following GDH (baseline 250.49 ± 95.65 *vs* post‐GDH 178.37 ± 88.15; T (42) = 6.50, *P* < 0.0001; Fig. 2).

### Quality‐of‐life scores

**Figure 3:**
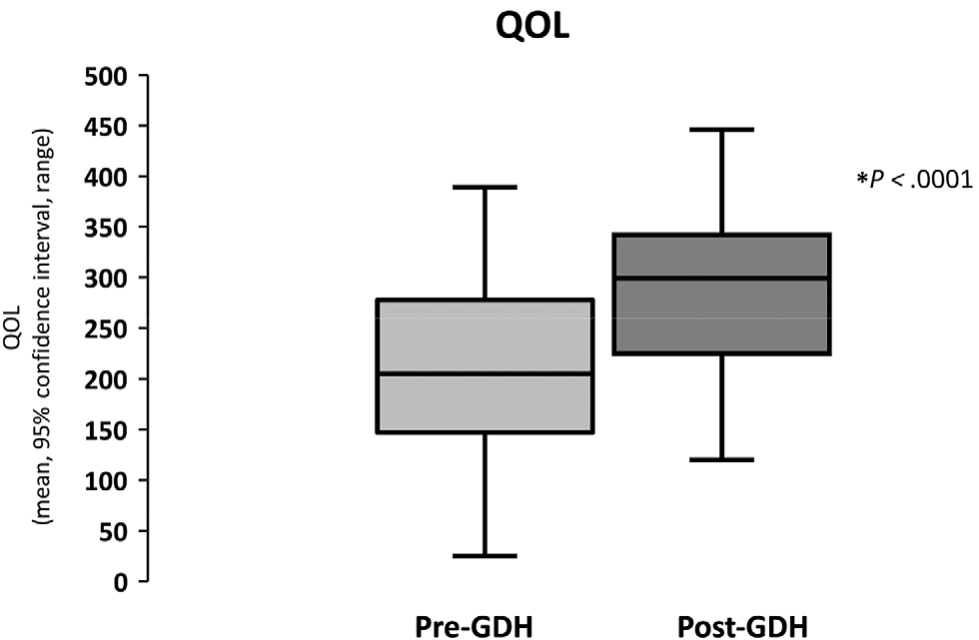
Improvement in quality‐of‐life (QOL) post‐gut‐directed hypnotherapy (GDH).

Following GDH, there were highly significant improvements in patients' ability to cope with problems, confidence and security, quality of sleep, physical well‐being, hopefulness, enjoyment of life, helplessness, relationships with family, maintenance of friendships, inferiority, and enjoyment of leisure (Table 2). There was also an overall large improvement in QOL after GDH (pre‐GDH 208.12 ± 70.53 *vs* post‐GDH 285.84 ± 74.92; T (42) = 5.84, *P* < 0.0001; Fig. 3).

### Anxiety and depression

**Figure 4:**
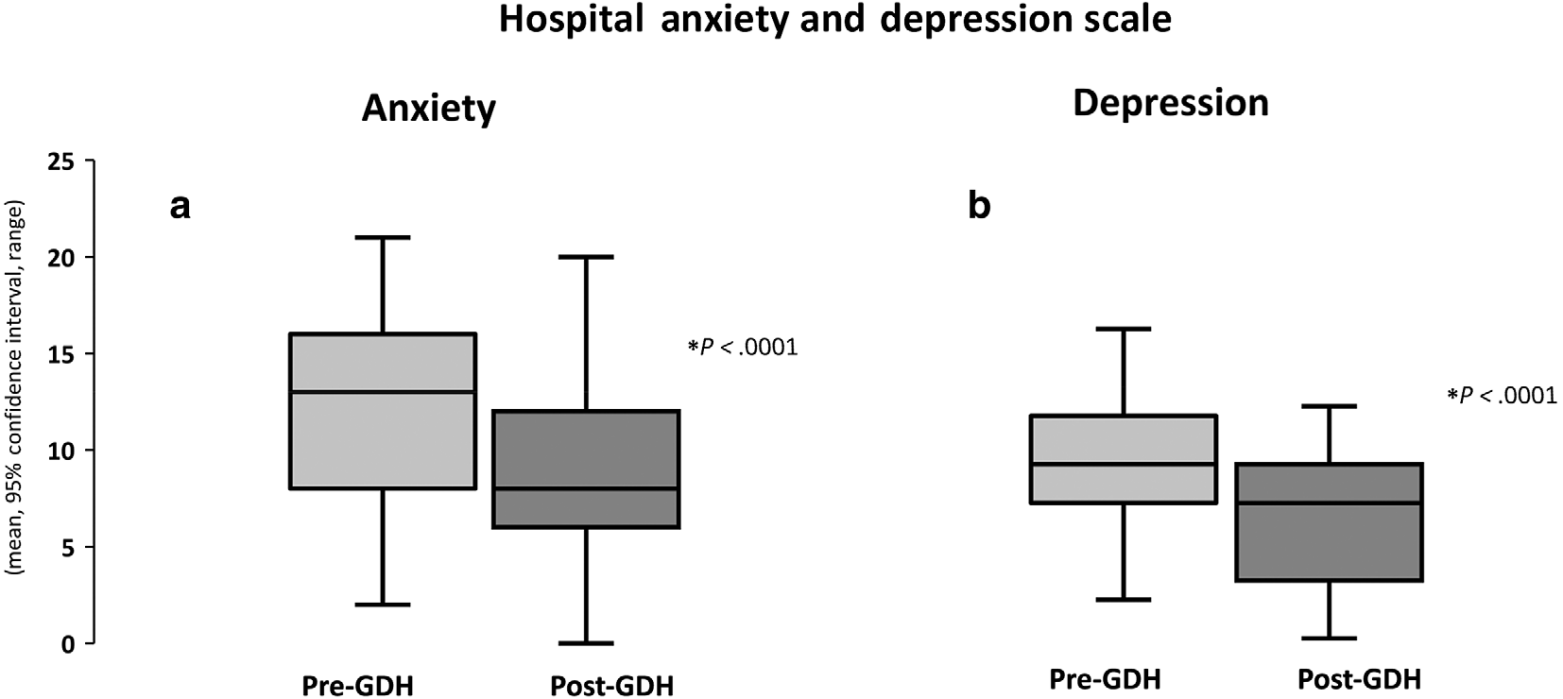
Improvements in hospital anxiety (a) and depression scale (b) post‐gut‐directed hypnotherapy (GDH).

Exactly 68% (30 of 44) of patients had a baseline HADS‐A score of ≥10, indicating clinical levels of anxiety, while 46% (20 of 44) of patients had a HADS‐D score of ≥10. Following GDH, there was a significant reduction in anxiety (pre‐GDH 12.64 ± 4.57 *vs* post‐GDH 9.22 ± 4.47; T (42) = 5.90, *P* < 0.0001; Fig. 4a) and depression scores (pre‐GDH 9.24 ± 3.14 *vs* post‐GDH 6.72 ± 3.64; T (42) = 5.77, *P* < 0.0001; Fig. 4b).

### Predictors of response to GDH defined by 50‐point improvement in IBS‐SSS

Overall, only seven patients did not respond to GDH.

Response rates to GDH did not differ by IBS‐subtype (IBS‐D 17/22 *vs* 20/22 IBS‐C and IBS‐M, *χ* ^2^ = 0.68, *P* = 0.41).

There was also no difference in median baseline HADS‐A (13 *vs* 15, *U* = 108.5, *P* = 0.51) and HADS‐D scores (9 *vs* 12, *U* = 84.5, *P* = 0.18), and median baseline non‐colonic symptom scores (242 *vs* 288.5, *U* = 136.50, *P* = 0.451) between responders and nonresponders.

Where the country of birth was known, response rates to GDH did not differ between those who were first‐generation British Asians compared with those who were born in the United Kingdom (first generation 9/11 *vs* 20/21 born in United Kingdom, Fishers exact test two‐tailed *P* = 0.30).

There was also no difference in the clinical response rates between British Asians of Indian (12 of 15 [80%]) and Pakistani descent (23 of 25 [90%]), Fisher's exact test two‐tailed *P* = 0.32.

Outcomes were also similar, regardless of the ethnicity of the therapist, with 21 of 23 (91%) patients treated by an Asian hypnotherapist responding, compared with 16 of 21 (76%) patients treated by a non‐Asian hypnotherapist (Fisher's exact test, two‐tailed *P* = 0.206).

## Discussion

This study is the first to evaluate the efficacy of GDH in an Asian population with refractory IBS. The findings that Asian patients were highly responsive to this form of therapy with significant improvements in all outcome measures, including gastrointestinal, extra‐intestinal, psychological symptoms, and quality of life, suggests that the development of this form of therapy may be helpful in Asian countries and merits further exploration.

The observed response rates to GDH in this population in improving IBS symptoms, QOL, anxiety, and depression were similar to previously reported improvements from our research group^27^, ^28^, ^39^ and others.^41^ This remained true with regards to GDH response rates in British Asians of Indian and Pakistani heritage.

Indeed, the magnitude of the mean reductions in IBS‐SSS following GDH were almost identical when compared with the improvements reported in the largest ever series and the largest clinical trial, using the same well‐established hypnotherapy protocol, incorporating outcomes from over 1400 patients^27^, ^37^ and in a smaller study in children and adolescents.^28^ With respect to extra‐intestinal symptoms, psychological symptom scores, and QOL, the improvements observed were also similar to our previous studies in other populations using the same validated outcome measures.^27^, ^28^, ^37^, ^38^ The observed improvement in often debilitating extra‐intestinal symptoms is important as these symptoms seldom respond to conventional, biological therapies targeting gastrointestinal symptoms, but have consistently been shown to respond to GDH.^25^

Although there was a slight numerical difference in the proportion of patients of Indian and Pakistani heritage that responded to GDH, there was no statistically significant difference between the groups. This suggests GDH is highly effective in Asians irrespective of specific cultural heritage and is similarly effective in other populations studied. Despite yoga originating in India, and sharing similarities with aspects of GDH^25^, ^42^ with some evidence for its effectiveness in treating musculoskeletal^43^ and abdominal pain,^44^ surprisingly, there was no evidence that patients of Indian origin were more receptive to this form of therapy. However, an explanation for this may be found in the fact that studies have shown the uptake of people practicing yoga in India is actually quite low (16.9%)^45^ and the uptake (0.46%) is even lower in UK populations.^46^ No equivalent studies have been performed in Pakistan. Hence in all countries in which studies have been performed, the majority of people do not practice yoga. Furthermore, no studies have been performed on the prevalence of yoga awareness (and how it may differ from the practice of yoga) in Asian countries or populations with Asian heritage in the West. In addition, this study was not designed to assess the proportion of patients of different ethnic backgrounds who would accept GDH as an additional treatment for their refractory IBS. It may be that future studies show differences emerging, which may be congruent with cultural awareness and meditative practices.

There was no difference in the response to GDH rates between patients seen by Asian and non‐Asian therapists. However, it must be noted that despite the lack of statistical significance, there was a moderate numerical difference implying that differences may emerge between these subgroups in future larger studies. Indeed, studies in IBS have shown that patient satisfaction is improved by shared cultural background between patient and clinician.^47^ This is thought to reflect intra‐cultural ease in therapeutic communication^47^ and should prompt increased cultural awareness when communicating with patients for clinicians practicing in multi‐cultural environments.^15^

There were several limitations to our study. Firstly, ours is a study with a relatively small number of participants in the area of GDH. Therefore, although our findings are highly significant and consistent with our experience in other populations, our data and experience will be important in the design of future larger studies to further explore inter‐ethnic differences in GDH effectiveness. Secondly, this was a study of GDH effectiveness in routine clinical practice, therefore there was no control group. However, this factor is mitigated to some extent by the fact that the results were almost identical to controlled trials in large numbers of patients using the same outcome measures.^27^ Finally, this is a study of outcomes in patients of Asian origin living in the United Kingdom, and our study did not prospectively examine the extent to which our patients' cultural and lifestyle practices have changed in comparison to those with IBS in their native countries. Indeed, studies have shown that with respect to cultural practices and views of health and disease, there are similarities, but also key differences between populations with different ethnic heritages in the West and in their respective countries of heritage.^48^, ^49^ However, data were available on the country of birth for the majority our participants. Interestingly, there were no differences in the response rates to GDH between those known to be first‐generation British Asians and those who were not first‐generation Asians. This finding suggests that those who may be closest in terms of cultural and lifestyle practices to their native countries are equally responsive to this form of treatment, further suggesting that GDH may be effective in Asian countries. Overall, our findings should therefore enhance our understanding of GDH as it applies to different populations worldwide and underscore the need for future large‐scale studies of this nature in Asian countries to confirm these findings.

In conclusion, we have shown for the first time that GDH is a highly effective treatment approach in an Asian population with refractory IBS. It significantly improves gastrointestinal, extra‐intestinal, psychological symptoms, and QOL. In light of this, to prevent ongoing disparities in the approach to treatment, it should be considered to be a standard part of the treatment pathway for patients with IBS on a global basis. These data have highlighted the need for developing services and population‐specific clinical studies to confirm these findings in Asian countries.

## Data Availability

Data are available upon reasonable request to the corresponding author.
